# Pharmacokinetic barriers to oxfendazole flukicidal efficacy

**DOI:** 10.1371/journal.pone.0355551

**Published:** 2026-08-25

**Authors:** Laura Ceballos, Candela Canton, Valeria Gayo, Laura Moreno, Carlos Lanusse, Luis Alvarez

**Affiliations:** 1 Laboratorio de Farmacología Centro de Investigación Veterinaria de Tandil (CIVETAN), CONICET-UNCPBA-CICPBA, Facultad de Ciencias Veterinarias, UNCPBA, Campus Universitario, Tandil, Argentina; 2 Instituto DILAVE, ‘Miguel C. Rubino’, Montevideo, Uruguay; National University of Rosario, ARGENTINA

## Abstract

Although oxfendazole (OFZ) has been traditionally used to control nematode parasites in domestic animals and more recently in human medicine, high efficacy against porcine cysticercosis and fasciolosis in both sheep and pigs has been observed when the drug is administered at higher dose levels. In this context, the plasma disposition kinetics and the pattern of drug accumulation in adult *Fasciola hepatica* was characterized in infected sheep orally treated with OFZ at either the nematodicidal dose of 5 mg/kg (OFZ_5_) or at a higher dose of 30 mg/kg (OFZ_30_)*.* OFZ was the main analyte detected in plasma of treated sheep. The systemic exposure (AUC_0–LOQ_) increased from 17.9 ± 3.71 µg.h/mL at the therapeutic dose to 85.4 ± 22.6 µg.h/mL with the highest dose treatment. The plasma Cmax value was approximately fourfold higher in the OFZ_30_ compared to the OFZ_5_ treated animals. The dose-related marked differences in OFZ systemic exposure were reflected in the accumulation into *F. hepatica* specimens, which was 332% higher in the OFZ_30_ group (4.28 µg/g) than in the OFZ_5_ group (0.99 µg/g). The data shown here demonstrate that increasing the OFZ dose is associated with enhanced plasma drug exposure and greater accumulation in the target trematode parasite, which helps to explain the enhanced efficacy of OFZ against adult liver flukes at the 30 mg/kg dose.

## 1. Introduction

Fasciolosis, caused by the trematode *Fasciola hepatica,* is a disease that affects domestic ruminants, including sheep, cattle, buffalo and goats. *F. hepatica* infection significantly impacts farm productivity by reducing weight gain, fertility, feed conversion efficiency and milk production as well as causing anemia and a decrease in work capacity [1]. Fasciolosis is also an important zoonosis. Nowadays, it is included by WHO in the list of the Neglected Tropical Diseases (NTDs), among the group of food borne trematodiases [2]. Human cases of fascioliasis are increasing with an estimation that at least 2.4 million people worldwide. No continent is free from the disease, and it is likely that areas with reported animal cases also have human cases [3]. Domestic animals become infected by eating metacercariae-contaminated grass, while humans are infected through the ingestion of contaminated edible vegetation or by drinking metacercariae-contaminated water [4]. The One Health approach recognizes the interconnectedness of human, animal, and environmental health, emphasizing the need for multidisciplinary strategies to prevent and control zoonotic diseases.

Chemotherapy remains the most effective strategy for controlling fasciolosis in animals, primarily through the use of anthelmintic drugs, including benzimidazole (BZD) derivatives, such as the halogenated thiol triclabendazole (TCBZ) and the methylcarbamate-BZD albendazole (ABZ), as well as other compounds like nitroxynil, closantel, and clorsulon [5,6]. Among all flukicides, TCBZ is the most widely used due to its high efficacy (>98%) against adult, early immature, and immature flukes [7]. The excessive reliance on chemical control has led to the selection of flukes resistant to commonly used drugs, such as TCBZ over the years [8–13] and ABZ, which is a growing challenge to both animal and human health [14–17]. The treatment of human fascioliasis relies almost exclusively on TCBZ [18]; however, its efficacy may vary depending on the susceptibility of the parasite isolate. Reduced efficacy of TCBZ in humans has recently been reported in disease-endemic communities [19–22]. This observation clearly exemplifies the selection of drug-resistant organisms by farming practices and their subsequent transmission to humans, underscoring the critical need to analyze liver fluke control within the One Health framework.

Because the development of new drugs involves multiple challenges -including timelines that often exceed a decade, success rates below 10%, and the high costs associated with the clinical trials required for approval- the strategy of drug re-evaluating has become increasingly relevant. This approach has been shown to increase success rates, reduce development costs, shorten time to market, and ultimately decrease the overall development risk compared with traditional drug-development pathways [23].

Oxfendazole (OFZ) is the active sulphoxide metabolite of fenbendazole (FBZ). The sulphoxidation is a rapid and reversible process which equilibrates with the respective thioether [24], although the equilibrium favors metabolism towards sulphoxidation. OFZ also undergoes a second, slower and irreversible oxidative step, resulting in the formation of the inactive metabolite fenbendazole sulfone (FBZSO_2_) [24]. Additionally, the metabolic sulphoreduction of OFZ to form the parent thioether (FBZ) has been shown to occur in ruminal and intestinal fluid contents from sheep and cattle [25]. OFZ and FBZ, like other BZD methylcarbamate such as ABZ or ABZSO exhibit high efficacy against lungworms and adult gastrointestinal nematodes, and effectively kill their larval stages. These compounds are commonly used for antiparasitic control in ruminants at doses ranging from 4.5 to 10 mg/kg, primarily targeting nematodes and tapeworms. However, while ABZ shows activity against adult liver flukes (>12–14 weeks old), OFZ/FBZ have shown little to no activity against *F. hepatica* [5]. In fact, Furmaga et al. [26] have reported efficacies of 14% and 20%, against liver fluke infections in sheep treated with OFZ at 5 and 15 mg/kg, respectively. The differential flukicidal effect observed between ABZ/ABZSO and FBZ/OFZ can be explained by differences in affinity for ß-tubulin (pharmacodynamics, PD) or in different amounts of drug reaching the target parasite (pharmacokinetics, PK). Supporting a PK-based limitation, lower peak plasma concentrations of OFZ (0.36 ± 0.05 µg/mL) were reported after FBZ administration (5 mg/kg) in sheep compared to the ABZSO concentrations (1.90 ± 0.11 µg/mL) achieved after ABZ treatment at the same dose [27]. Given that oral ingestion is the primary route of *in vivo* drug entry into adult liver flukes [28–30], variations in systemic drug exposure likely account for the observed differences in efficacy. Additional indirect support for this PK hypothesis comes from studies showing a clear dose exposure-efficacy relationship for OFZ. Although performed in different host species and against parasites with distinct anatomical locations, administration of a single high oral dose of 30 mg/kg OFZ achieved 100% efficacy against naturally occurring gastrointestinal nematodes in pigs, including *Ascaris suum*, *Oesophagostomum* spp., *Metastrongylus* spp., and *Trichuris suis* [31], as well as against porcine cysticercosis [32,33]. Although the parasite species and host differ from those evaluated in the present study, these findings provide indirect evidence that insufficient systemic availability at the conventional dose may explain the limited flukicidal activity of OFZ against *F. hepatica*. Furthermore, when the same high dose was experimentally used in naturally infected animals, efficacy against adult *F. hepatica* was demonstrated in both sheep [34] and pigs [35]. Those results seem to discard a PD- limitation (i.e., poor OFZ receptor affinity in the trematode parasite) and instead support a PK-based restriction affecting drug efficacy.

Based on this hypothesis, the main objectives of the current work were a) to characterize the plasma PK of OFZ and its metabolites in healthy sheep following administration at either the standard nematodicidal dose (5 mg/kg) or an elevated experimental dose (30 mg/kg), and b) to perform a drug tissue distribution assessment to characterize *in vivo* the dose-related OFZ/metabolites distribution patterns within adult *F. hepatica* recovered from artificially-infected sheep, treated at the mentioned dosages.

## 2. Materials and methods

### Experimental design

**Animals:** the PK and tissue distribution studies were conducted on healthy pre-dewormed Corriedale sheep. The animals came from an area free of *F. hepatica*. Likewise, the absence of liver fluke infection was individually confirmed prior to the treatment by analyzing *F. hepatica* eggs in fecal samples, following routine laboratory methods adapted from [36,37]. The animals were housed indoors with water provided *ad libitum* and were fed with commercial balanced diet (*Tandilcoop ovinos*, Cooperativa Agropecuaria de Tandil, Tandil, Argentina). Food and water intake were monitored daily throughout the experimental period. Feed was offered in measured amounts. Animals were observed daily by trained veterinary personnel for any changes in clinical signs or feeding behavior. Animal procedures and management protocols were carried out in accordance with the Animal Welfare Policy (Act 087/02) of the Faculty of Veterinary Medicine, Universidad Nacional del Centro de la Provincia de Buenos Aires (UNCPBA), Tandil, Argentina, and internationally accepted animal welfare guidelines (AVMA, 2020). The experimental protocol (Plasma pharmacokinetics and tissue distribution of oxfendazole in sheep, following administration at doses of 5 and 30 mg/kg) was approved under **Protocol No. 4/24**. No alternative study models to the use of experimental animals exist to carry out the studies proposed in this work, as the assessment of oxfendazole tissue distribution requires the collection of organ samples (liver, bile and parasites), a terminal procedure that cannot be replaced by non-invasive methods**.** Consequently, euthanasia was performed by trained veterinarians using a captive bolt followed by exsanguination, strictly adhering to animal welfare regulations to ensure a humane endpoint.

***PK study:*** Twelve (12) sheep (aged 8–9 months, 24–35 kg body weight) were used. After being fed, the animals were assigned into two experimental groups of 6 animals each and orally treated with OFZ (Synanthic^®^ 9.06%, Merial, France) at either 5 (OFZ_5_) or 30 (OFZ_30_) mg/kg. Blood samples were taken from each animal from the jugular vein in Vacutainer® tubes (Becton Dickinson, NJ, USA) at: 0, 1, 3, 6, 9, 12, 18, 24, 28, 32, 48, 52, 56, 74, 80 and 96 h post-treatment (p.t.). Plasma was separated by centrifugation at 3000 *g* for 15 min, placed into plastic tubes and frozen at −20ºC until analysis by High Performance Liquid Chromatography (HPLC).

***Tissue distribution study*:** This assay was conducted two weeks after the end of the PK trial. Nine (9) *F. hepatica*-free sheep (aged 9 months, 26–35 kg body weight) were included in the study. Animals were orally infected with seventy-five (75) *F. hepatica* metacercariae (mt). These were obtained under laboratory conditions by infecting *Lymnaea neotropica* snails with *F. hepatica* eggs from untreated sheep (Centro de Diagnóstico e Investigaciones Veterinarias (CEDIVE), Facultad de Ciencias Veterinarias, Universidad Nacional de La Plata, Chascomús, Argentina), and characterized by Egg Hatch Test as ABZ-susceptible. Sixteen (16) weeks after infection, the animals were randomly allocated into three groups: the OFZ_5_ group (n = 4), orally treated with OFZ at 5 mg/kg (nematicidal dose); the OFZ_30_ group (n = 4), orally treated with OFZ at 30 mg/kg; and the Control group (n = 1), which consisted in an untreated animal used that was used for matrix blanking and infection verification, and not as a baseline for statistical concentration comparisons. All experimental animals were sacrificed at 12 (OFZ_5_) or 24 (OFZ_30_) h p.t. These sacrifice times were coincident with the C_max_ value obtained in the PK study. The animal in the Control group was sacrificed concurrently with the animals in Group OFZ_30_.

Samples of blood (collected into heparinized tubes), liver, bile, and *F. hepatica* specimens were obtained from the three experimental groups. The blood samples were centrifuged to obtain plasma. Adult *F. hepatica* specimens were recovered from all animals as previously described by Ceballos et al. [30]. Briefly, at necropsy, the gall bladder and liver of each sheep were examined for the presence of live and dead *F. hepatica*. After incising the gall bladder and bile ducts, the liver was cut along the large and small bile ducts and hepatic veins and carefully searched for flukes. Manual pressure was applied, primarily using the thumbs in a distal-to-proximal direction along the bile ducts, to facilitate the expulsion of liver flukes. The livers were then cut into thin slices (0.5–1.0 cm in width) and incubated in warm (37 °C) saline solution to enhance the recovery of *F. hepatica* specimens (both mature and immature forms). The individual number of parasites recovered from each animal was recorded. All samples obtained from treated and untreated experimental groups were stored at −20 °C until analysis of OFZ and its metabolites by high-performance liquid chromatography (HPLC).

### Analytical procedures

#### Chemicals.

OFZ and its metabolites, FBZ and FBZSO_2_, pure reference standards (99% purity), were purchased from Toronto Chemicals Research Inc. (Toronto, Canada). Albendazole sulphoxide (ABZSO,) used as internal standard (IS, 99% purity) was purchased from (Toronto Research Chemicals), high-purity water was obtained from a Milli-Q water purification system (Simplicity®, Millipore, Brazil). The analytical standard solutions were prepared in methanol (MeOH), and maintained at −20 °C throughout the duration of the study to ensure stability.

#### Analytical phase.

Full validation of the analytical procedures for the extraction and quantification of each molecule (OFZ, FBZ, FBZSO_2_ and ABZSO) in each biological matrix was performed before the analysis of the experimental samples, according to the ICH Q2(R1) (ICH Harmonised Guideline, 2005). The calibration curves and quality control (QC) samples were prepared with drug-free plasma and tissues/fluids (*F. hepatica*, bile and liver). The linearity was tested by constructing calibration curves for each compound in tissues (*F. hepatica*, bile and liver) and plasma.

The calibration ranges for OFZ, FBZ, FBZSO_2_ were the following: for plasma 0.05–4 µg/mL; for *F. hepatica* 0.25–5 µg/g, for bile 0.2–5 µg/mL, for liver 0.2–5, 5–15 µg/g, using 6 different concentrations (n = 3, for each point of concentration). The data were analysed for linearity using the least-squares regression method, using the Run Test and ANOVA to determine if the data differed from a straight line.

The Limit of Detection (LOD) was estimated by integrating the baseline noise of the HPLC system in the area covering the mean retention time (RT) of each analyte and was defined as the mean baseline noise/IS peak area ratio plus three standard deviations (SD). The Limit of Quantification (LOQ) was defined as the lowest drug concentration (n = 6) on each tissue standard curve that could be quantified with a precision not exceeding 20% and accuracy within 20% of the nominal concentration. Resulting in an LOD of ≤0.02 µg/mL for OFZ and its metabolites in plasma. The LOQ values obtained for OFZ and its metabolites were as follows: plasma, 0.05 µg/mL; liver, 0.2 µg/g, bile, 0.2 µg/mL; and *F. hepatica,* 0.25 µg/g. Values in plasma below the LOQ were excluded from the PK analysis.

The absolute recovery of analytes from each matrix was calculated by comparing the peak areas of spiked experimental samples with those obtained from direct injections of standards in the mobile phase. Mean absolute recoveries were >70% in all cases, with coefficients of variation (CV) ranging from 5 to 10%. The LOQ was defined as the lowest measured concentration with a CV < 20%, accuracy of ± 20% and an absolute recovery of >70%.

#### Plasma and tissues samples process.

The preparation, clean up and physicochemical drug/metabolites extraction of the collected samples were as follows:

**Plasma samples:** Experimental and fortified plasma samples (500 µL), spiked with 20 µL (25 µg/mL) of ABZSO as IS, were mixed with 1 mL of acetonitrile and agitated for 15 minutes using a multitube vortexer (VWR Scientific Products, West Chester, PA, USA). The samples were then centrifuged at 4ºC for 10 minutes at 2000 g (Jouan®, BR 4i Centrifuge, Saint Herblain, France). The clear supernatant was transferred manually to a 5 mL glass tube, and the precipitates obtained were re-extracted with 1 mL of acetonitrile following the same procedure described above. The supernatants were evaporated to dryness in a vacuum concentrator (Speed-Vac®, Savant, USA). The dried extract was reconstituted with 250 µL of mobile phase (ACN/water, 27/73) and shaken vigorously for 5 minutes. A 50 µL aliquot from each individual sample (one sample per animal per time point) was injected into the HPLC system.

**Liver samples:** OFZ and its metabolites, FBZSO_2_ and FBZ, were extracted from both experimental and fortified liver samples, following the methodology described by Ceballos et al., [30]. Briefly, 0.5 g of minced liver samples were spiked with the IS (ABZSO, 40 µL of 25 µg/mL solution). After 5 minutes, 0.5 mL of NaOH (1 N) and 1.5 mL of acetonitrile were added, and the samples were agitated for 15 minutes using a high-speed vortex shaker. After mixing, the samples were sonicated for 10 minutes and then centrifuged at 4ºC for 15 minutes at 2000 g. The clear supernatant was transferred to a new tube, and the extraction procedure was repeated. The collected supernatant was concentrated to dryness in a vacuum concentrator, then reconstituted with 0.25 mL of mobile phase (ACN/water, 27/73) and vigorously shaken for 5 minutes. A 50 µL aliquot of the reconstituted sample was injected into the chromatographic system.

**Bile samples:** Bile samples (500 µL), both experimental and fortified, were spiked with IS (40 µL of 25 µg/mL solution) and then extracted by adding 1.5 mL of ethyl acetate (twice). After shaking (multivortex 50 min), and centrifugation (2000 g, 15 min, 4ºC), the clear supernatant (ethyl acetate phase, 3 mL) was concentrated to dryness using a vacuum concentrator and then reconstituted with 250 µL of mobile phase (ACN/water, 27/73) and vigorously shaken (5 min). Fifty (50) µL of the reconstituted volume was injected into the chromatographic system.

### *Fasciola hepatica* specimens

Experimental and fortified *F. hepatica* samples were processed following the methodology described by Mottier et al. [38]. Homogenized parasite material (0.1 g) was spiked with IS (ABZSO 20 µL of 25 µg/mL solution). After 5 minutes, the samples were mixed with 1.5 mL of acetonitrile, agitated for 5 minutes using a multi-vortex and then centrifuged at 2000 g for 10 minutes at 10ºC. The supernatant was separated and the extraction procedure was repeated three times. The final collected acetonitrile phase (4.5 mL) was concentrated to dryness, reconstituted with 150 µL of mobile phase (ACN/water: 27/73) and thereafter shaken for 5 minutes. A 50 µL aliquot of each solution was injected into the chromatographic system.

### HPLC system and chromatography

After extraction, 50 µL of the sample of each matrix was injected into a Shimadzu Chromatography System (Shimadzu Corporation, Kyoto, Japan) using a gradient pump, a UV detector set at 292 nm. HPLC equipment composition and settings were described by Moreno et al [39], elution from the stationary phase was carried out at a flow rate of 1.2 mL/min using a mobile phase based on acetonitrile and ammonium acetate buffer (0.025 M, pH 6.6). The C18 reversed-phase column (5 µm, 250 mm × 4.6 mm) was Kromasil (Kromasil®, Sweden).

### Pharmacokinetic analysis of the data

Non-compartmental PK analysis of the plasma concentration versus time curves for OFZ and its metabolites in each individual animal after the different treatments was performed using the PK Solution 2.0 (Summit research services, CO, USA). The peak concentration (C_max_) and time to peak concentration (T_max_) were determined from the plotted concentration–time curve of each analyte. The area under the concentration–time curve from zero up to the limit of quantification (AUC_0-LOQ_) was calculated by using the trapezoidal rule [40]. The terminal elimination rate constant (β) was determined by linear regression analysis of the terminal log-linear phase of the concentration–time profile. AUC_0-LOQ_ was further extrapolated to infinity (AUC_0-∞_) by dividing the last experimental concentration by β. The elimination (T½_el_) and absorption half-life (T½_ab_) were calculated using the formulas ln 2/β and ln 2/k, respectively. The mean residence time (MRT) was determined as AUMC/AUC_0-LOQ_ [41], where AUMC represents the area under the curve of the product of time and the plasma drug concentration versus time from zero to infinity [40], and AUC_0-LOQ_ is as described above.

The PK parameters and concentration data are expressed as arithmetic mean ± SD. The AUC value was considered an indicator of the total drug exposure. The mean PK parameters for OFZ and its metabolites were statistically compared using Student´s *t-*test, with a significance level of P < 0.05. Statistical analysis was performed using Instat 3.0 software (GraphPad Software, CA, USA).

## 3. Results

**Fig 1:**
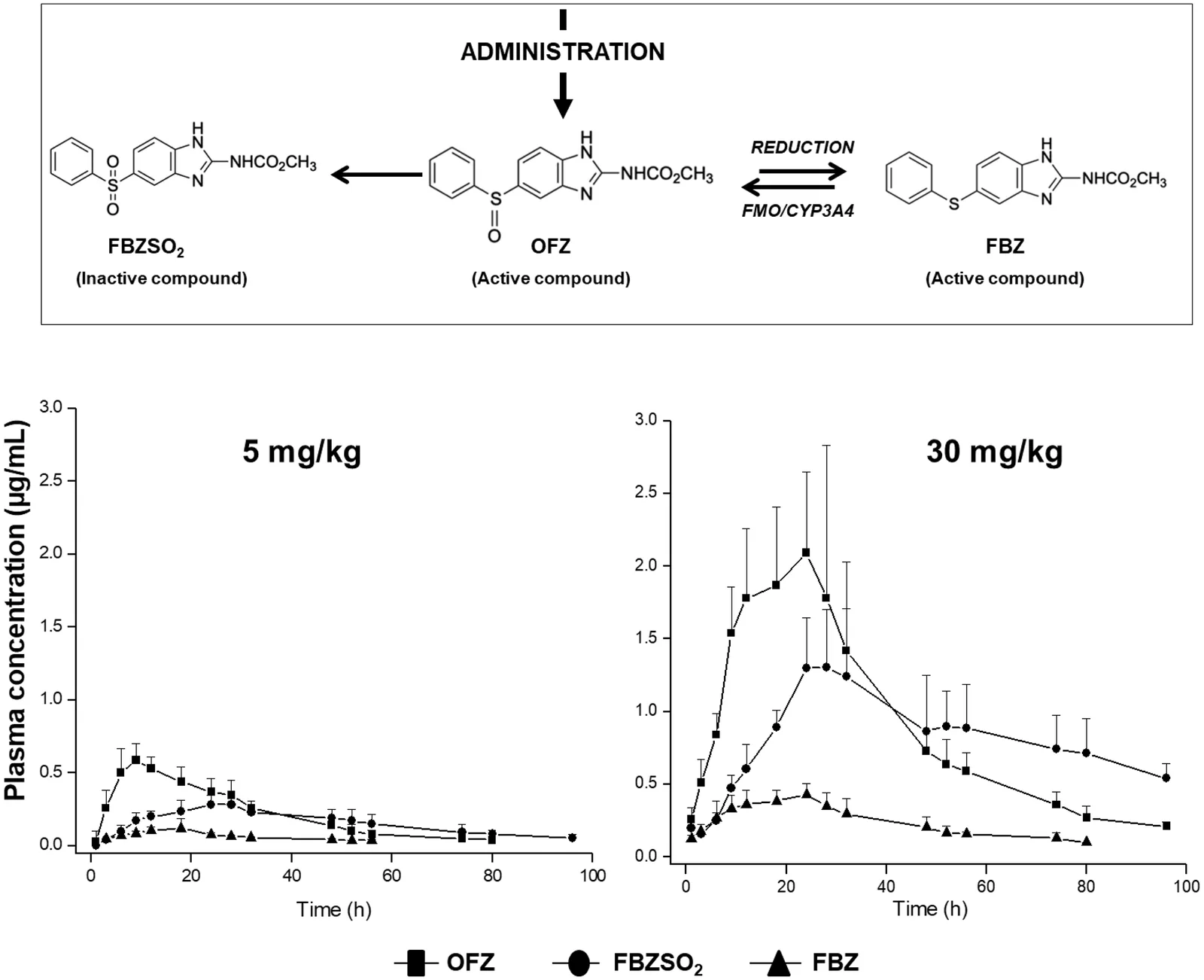
Comparative (mean ± SD) plasma concentration profiles of oxfendazole (OFZ), fenbendazole sulphone (FBZSO_2_) and fenbendazole (FBZ) obtained after OFZ oral administration to sheep at 5 (OFZ_5_, n = 6) or 30 mg/kg (OFZ_30_, n = 6). ■ OFZ, ● FBZSO_2_, ▲ FBZ.

None of the animals involved in the different studies showed any adverse events (such as changes in food and water consumption, altered basic clinical signs, etc.), even after the dose of 30 mg/kg. Following the administration of OFZ to sheep, OFZ and its metabolites, the inactive FBZSO_2_ and the active FBZ, were detected in plasma samples. Fig 1 illustrates the effect of the dose rate on the plasma disposition of OFZ and its metabolites. OFZ was the main analyte quantified in plasma from 1 up to 96 h post-treatment, achieving a Cmax of 0.64 ± 0.12 (OFZ_5_) and 2.47 ± 0.64 (OFZ_30_) obtained at 9.50 ± 2.26 h and 19.7 ± 6.74 h (Tmax), respectively. FBZ concentrations were much lower than OFZ or FBZSO_2_, and were detected up to 56 h (OFZ_5_) and 80 h (OFZ_30_) post-treatment. The plasma disposition kinetics data for OFZ and its metabolites in both treated groups are summarized in Table 1.

**Table 1:** Plasma pharmacokinetic parameters (arithmetic mean ± SD) for oxfendazole (OFZ), fenbendazole sulphone (FBZSO_2_) and fenbendazole (FBZ), obtained after the oral administration of OFZ at 5 (OFZ_5_) or 30 (OFZ_30_) mg/kg to sheep.

Pharmacokinetic parameters	OFZ		FBZSO_2_		FBZ	
	OFZ_5_ _(5 mg/kg)_	OFZ_30_ _(30 mg/kg)_	OFZ_5_ _(5 mg/kg)_	OFZ_30_ _(30 mg/kg)_	OFZ_5_ _(5 mg/kg)_	OFZ_30_ _(30 mg/kg)_
Cmax (µg/mL)	0.64 ± 0.12	2.47 ± 0.64*	0.30 ± 0.07	1.42 ± 0.44*	0.14 ± 0.06	0.46 ± 0.07*
Tmax (h)	9.50 ± 2.26	19.7 ± 6.74*	26.0 ± 2.19	27.3 ± 3.93	14.0 ± 3.10	20.7 ± 6.89
AUC_0–LOQ_ (µg.h/mL)	17.9 ± 3.71	85.4 ± 22.6*	13.8 ± 3.75	75.6 ± 17.5*	3.12 ± 0.67	16.0 ± 6.06*
AUC_0-∞_ (µg.h/mL)	29.5 ± 7.56	165 ± 71.3*	35.3 ± 6.91	172 ± 72.8*	7.15 ± 1.74	31.8 ± 10.4*
T½el (h)	15.4 ± 2.02	21. 0 ± 3.44	25.3 ± 7.12	59.2 ± 29.6	16.3 ± 6.91	21.15 ± 5.08
T½ab (h)	2.8 ± 1.37	5. 46 ± 2.45	6.77 ± 1.60	6.76 ± 3.35	5.20 ± 3.07	4.73 ± 1.90
MRT (h)	25.3 ± 3.89	37.8 ± 4.88*	37.9 ± 6.94	78.3 ± 19.4*	20.8 ± 3.62	30.2 ± 10.9*

Cmax: peak plasma concentration; Tmax: time to the Cmax; AUC_0–LOQ_: area under the plasma concentration vs. time curve from zero up to the last quantifiable concentration; AUC_0–∞_: area under the concentration vs. time curve extrapolated to infinity; T½el- T½ab elimination/absorption half-life; MRT: mean residence time. * Indicates statistically significant differences (P < 0.05) between experimental groups.

Significant dose-dependent differences were observed for all three analytes, affecting both the mean plasma concentrations at the sampling times and the resulting PK parameters. Systemic exposure (expressed as AUC), peak plasma concentrations (C_max_), and mean residence time (MRT) were notably higher in animals receiving 30 mg/kg compared to those receiving 5 mg/kg. Specifically, increasing the dose from 5 to 30 mg/kg led to significant increases (P < 0.05) in both AUC_0–LOQ_ (from 17.9 ± 3.71 to 85.4 ± 22.6 µg·h/mL) and Cmax (from 0.64 ± 0.12 to 2.47 ± 0.64 µg/mL), a lack of dose proportionality was evident. The dose-adjusted C_max_ and AUC_0-LOQ_ values were only 64% and 79%, respectively, of those measured at the 5 mg/kg dose. The lack of dose proportionality observed for OFZ may be associated with a low dissolution rate after the higher OFZ dose. A similar up-trend was observed for both metabolites, FBZSO_2_ and FBZ. Moreover, the MRT for all analytes was significantly longer (P < 0.05) after the 30 mg/kg dose of OFZ.

**Fig 2:**
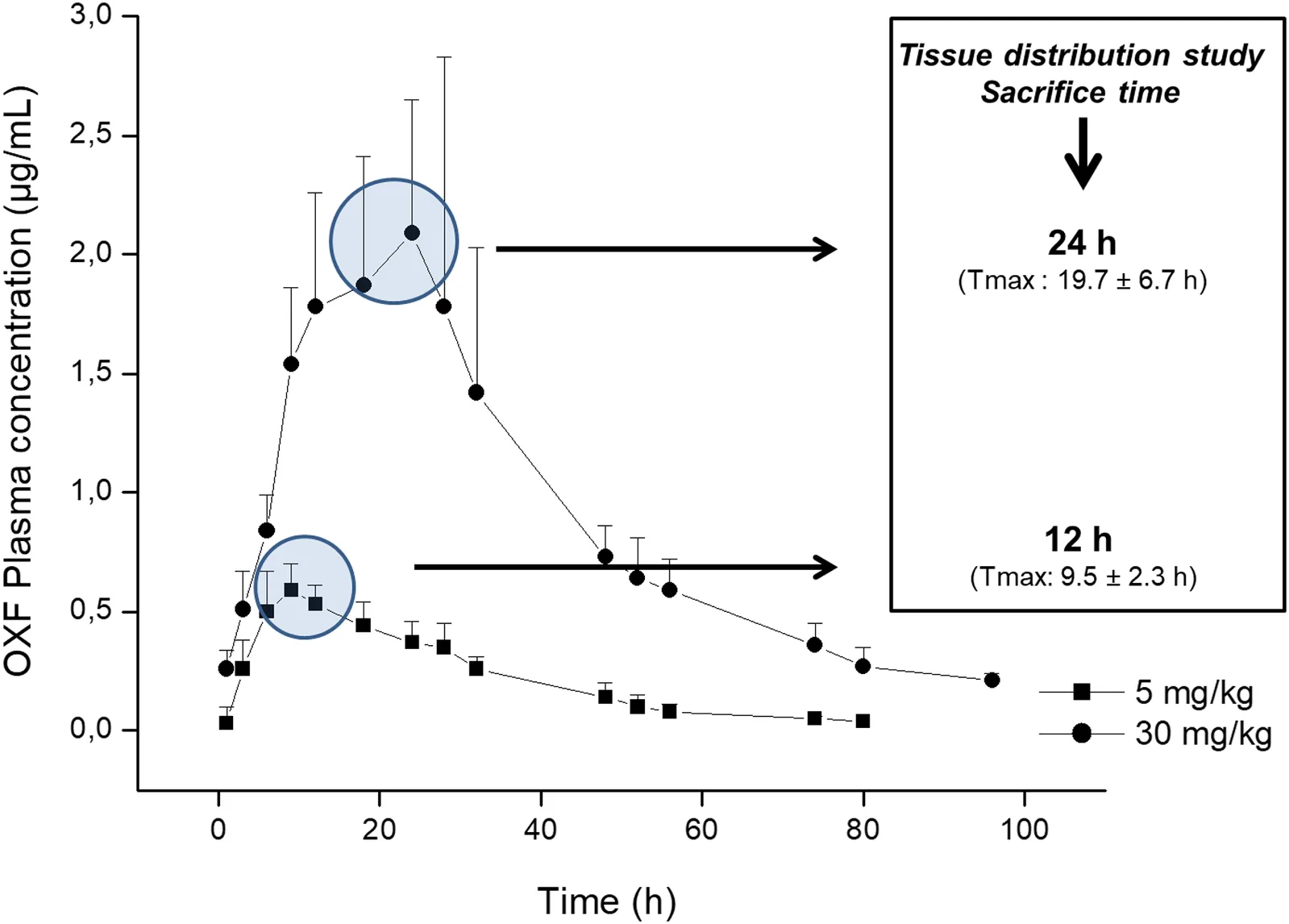
Comparative (mean ± SD) plasma concentration profile of oxfendazole (OFZ) obtained after its oral administration at 5 or 30 mg/kg to sheep. The graph illustrates (blue circles) the time to peak concentration (Tmax) achieved by each dose. This information was used to set the sacrifice times for the tissue distribution study, thereby ensuring parasite collection coincided with the peak drug availability in the host’s systemic circulation.

OFZ accounted for 51.4% (OFZ_5_) and 48.2% (OFZ_30_) of the total AUC_0-LOQ_, considering all detected analytes. The comparative plasma PK profile (mean ±SD) of this analyte is shown in Fig 2. OFZ was rapidly absorbed and detected in both experimental groups from the first sampling time (1 h), and remained detectable up to 80 (OFZ_5_) or 96 (OFZ_30_) h post-OFZ administration. Its plasma concentrations were consistently higher in OFZ_30_ group throughout the trial. The peak concentration in plasma was reached at 9.50 ± 2.26 h after administration of 5 mg/kg and was significantly (P < 0.05) delayed to 19.7 ± 6.74 h in the group receiving the higher dose. Based on these results, the accumulation study was defined at 12 h (OFZ_5_) and 24 h (OFZ_30_) after OFZ administration.

**Fig 3:**
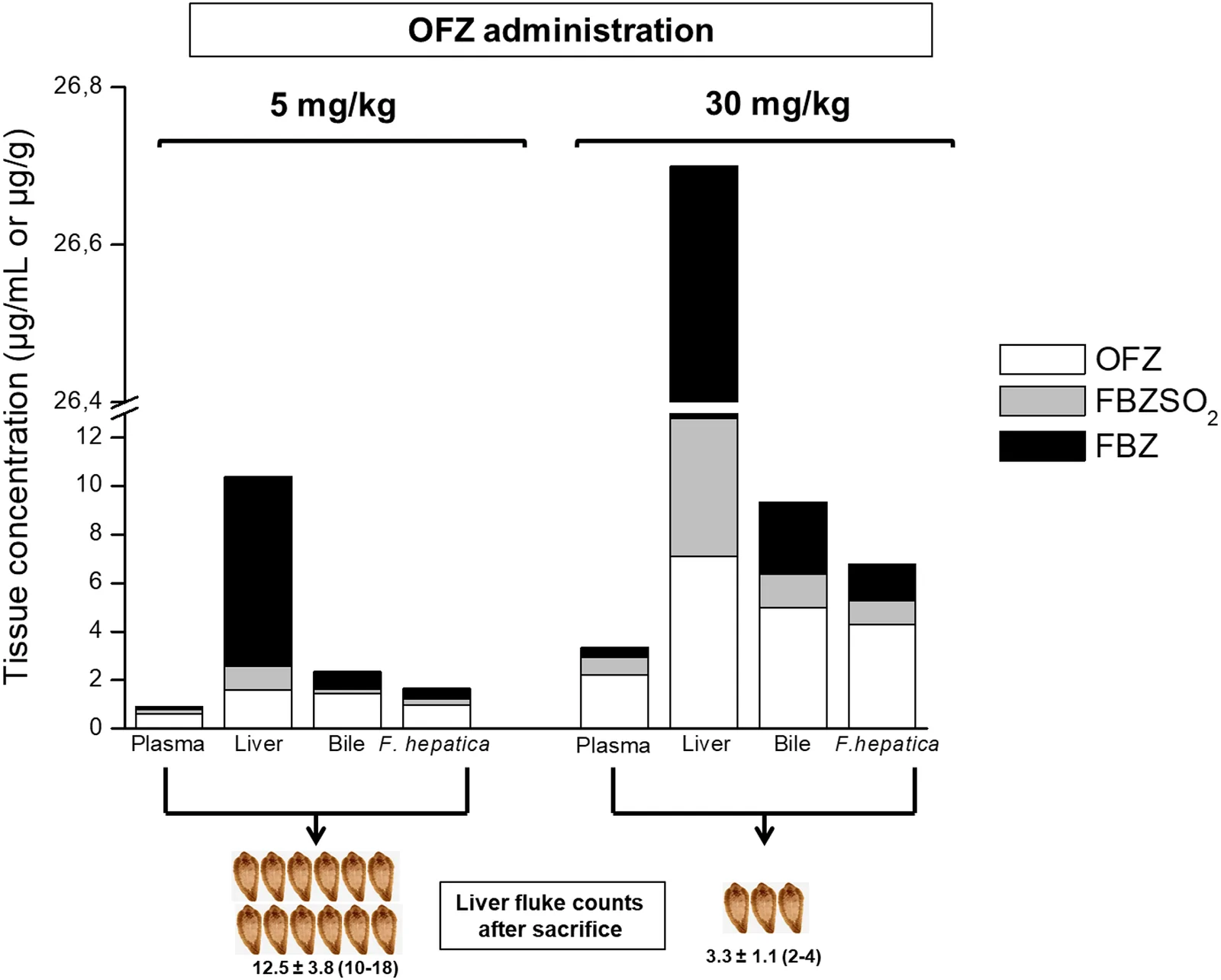
Comparative (mean ± SD) concentrations of oxfendazole (OFZ) and its metabolites fenbendazole sulphone (FBZSO_2_) and fenbendazole (FBZ) in plasma, liver, bile and mature liver flukes at the time of peak concentration after the oral administration of OFZ at 5 (12 h p.t.) or 30 mg/kg (24 h p.t.), to infected sheep. Additionally, the number of adult parasites (arithmetic mean ± SD) collected at sacrifice is shown.

Following treatment in the *Tissue Distribution Study*, animals were sacrificed at time points calculated to be 21–26% greater than the time of maximum plasma concentration (T_max_) for their respective dose, to better visualize the differences between groups. OFZ and its metabolites, FBZSO_2_ and FBZ, were measured in all matrices evaluated. Fig 3 shows the contribution of each analyte to the total amounts of drug recovered from plasma, liver, bile and adult specimens of *F. hepatica* after OFZ administration at both, 5 and 30 mg/kg. A similar profile was observed in both groups. OFZ represented more than 50% of the total drug detected in plasma, bile and parasites followed by FBZ and lowest concentrations of FBZSO_2_. In contrast, FBZ in liver accounted for more than 70% of total drug, reaching concentrations of 7.80 ± 1.80 µg/g and 13.9 ± 2.60 µg/g after OFZ administration at 5 or 30 mg/kg, respectively.

Table 2 summarizes the concentration of each analyte in the assessed fluid/tissues after OFZ administration at either 5 mg/kg or 30 mg/kg. Plasma concentrations at each sampling time were 0.59 ± 0.04 (12 h p.t., OFZ_5_) and 2.22 ± 0.58 (24 h p.t., OFZ_30_) µg/mL, which were similar (P > 0.05) to the C_max_ obtained in the PK study. Significant differences in drug accumulation were observed in all matrices analyzed between groups. The accumulation of OFZ in adult *F. hepatica* increased from 0.99 ± 0.20 µg/g (OFZ_5_) to 4.28 ± 1.4 µg/g (OFZ_30_), representing a 332% increase. Similarly, FBZ concentration in parasites were also 3.36 times higher in the OFZ_30_ group compared to the OFZ_5_ group. Moreover, the inactive metabolite FBZSO_2_ also showed a significant increase in parasite tissue (more than fourfold higher) following treatment with 30 mg/kg dose.

**Table 2:** Oxfendazole (OFZ), fenbendazole sulphone (FBZSO_2_) and fenbendazole (FBZ) concentrations (arithmetic mean ± SD) obtained in plasma, liver, bile and mature specimens of *Fasciola hepatica*, collected at the time of peak plasma concentration (Tmax) from sheep orally treated with OFZ at 5 (OFZ_5_) or 30 (OFZ_30_) mg/kg dose.

Sheep Tissue	OFZ		FBZSO_2_		FBZ	
	OFZ_5_ _(5 mg/kg)_	OFZ_30_ _(30 mg/kg)_	OFZ_5_ _(5 mg/kg)_	OFZ_30_ _(30 mg/kg)_	OFZ_5_ _(5 mg/kg)_	OFZ_30_ _(30 mg/kg)_
Plasma (µg/mL)	0.59 ± 0.04	2.22 ± 0.58*	0.19 ± 0.11	0.72 ± 0.20*	0.12 ± 0.01	0.39 ± 0.13*
Liver (µg/g)	1.60 ± 0.80	7.10 ± 2.19*	0.98 ± 0.30	5.70 ± 1.15*	7.80 ± 1.80	13.9 ± 2.60*
Bile (µg/mL)	1.46 ± 0.04	5.00 ± 3.42*	0.18 ± 0.04	1.36 ± 0.36*	0.71 ± 0.10	2.94 ± 2.26*
*F. hepatica* (µg/g)	0.99 ± 0.20	4.28 ± 1.40*	0.22 ± 0.06	1.01 ± 0.15*	0.44 ± 0.12	1.48 ± 0.32*

*Statistical differences in tissue concentration between experimental groups (P < 0.05).

Although this assay was not designed to evaluate clinical efficacy, a noticeable numerical difference in the individual number of adult parasites recovered at sacrifice was observed between treated groups. Fluke counts ranged from 10–18 specimens in the OFZ5 group (sacrificed at 12 h p.t.) compared to only 2–4 specimens in the OFZ30 group (sacrificed at 24 h p.t.), while 15 flukes were recovered from the single untreated control animal. However, no definitive efficacy conclusions can be drawn from these data due to two major methodological limitations: the sample size of the control untreated group and the non-equivalent post-treatment sampling times between groups, which implies that parasites in the high-dose group were exposed to the drug for a longer duration prior to sacrifice, which represents a confounding factor when interpreting the apparent drug-induced parasite reduction.

## 4. Discussion

Current strategies for controlling fasciolosis in ruminants rely primarily on the use of anthelmintic drugs. Given the increasing development of resistance to the most commonly used compounds, particularly TCBZ, and the shortage of new molecules, the re-evaluation (repurposing) of existing drugs becomes a worthwhile strategy. Ideally, alternative compounds should display a mechanism of action different from that of TCBZ in order to overcome or compensate for the loss of efficacy associated with TCBZ-resistant isolates. Benzimidazoles (BZDs) exert their anthelmintic activity by inhibiting microtubule assembly through binding to β-tubulin, leading to cytoskeletal disruption, impairment of organelle transport, and inhibition of cell division [42,43]. OFZ shares this mechanism of action with ABZ, another BZD methylcarbamate approved for use in human and veterinary medicine. However, published data indicate that OFZ inhibits helminth microtubule formation more selectively than mammalian tubulin, whereas ABZ affects both helminth and mammalian microtubules with similar potency [44,45].

In nematodes, resistance to BZDs has been strongly associated with reduced high-affinity binding to β-tubulin and specific point mutations at codons 200, 167, or 198 of the β-tubulin gene [46]. In contrast, in *F. hepatica*, the TCBZ-resistant phenotype does not appear to be consistently linked to the classical β-tubulin mutations described in nematodes [47]. Moreover, field reports have documented isolates exhibiting differential susceptibility patterns to ABZ and TCBZ (e.g., ABZ-susceptible but TCBZ-resistant, and vice versa) [15,48], suggesting that distinct mechanisms may underlie resistance to different BZD derivatives in *F. hepatica*. Taken together, these findings indicate that side-resistance among BZD compounds in *F. hepatica* is not necessarily predictable and may depend on the specific resistance mechanism involved. Therefore, despite sharing a common molecular target, OFZ could still represent a potential alternative for the control of TCBZ-resistant fluke populations.

Notably, OFZ is currently being evaluated for human parasite control [49], representing a further advantage. Recent studies have evaluated the safety and tolerance of increasing doses of OFZ in healthy human volunteers [50,51]. Other clinical trials are focusing on the use of different OFZ regimens in patients with mild parenchymal brain cysticercosis [52], and for human fascioliasis in endemic communities where triclabendazole resistance is a growing concern [53]. However, OFZ is not recommended as a flukicidal since the recommended OFZ dose for controlling gastrointestinal nematodes in ruminants (5 mg/kg), has been shown to be ineffective against *F. hepatica* [5,26], even though *in vitro* studies have confirmed its potential flukicidal effect [54]. This suggests a possible PK instead of PD limitation of the drug, preventing it from reaching and maintaining effective concentrations for a sufficient period to eliminate this trematode parasite. This hypothesis is further supported by studies in which the infection with *F. hepatica* was controlled both in sheep and pigs when OFZ was used at 30 mg/kg [34,35].

The current study was designed to test the hypothesis that the concentrations of OFZ and its metabolites at the site of parasite localization are critical for the elimination of adult flukes. Our findings at the 5 mg/kg dose, which showed plasma concentrations of OFZ and its FBZ metabolite consistent with previous reports [27,55,56], resulted in levels clearly insufficient to achieve clinical efficacy against *F. hepatica* in sheep [57]. In this context, ABZ serves as a relevant reference compound within the methylcarbamate group. While a 7.5 mg/kg dose of ABZ is traditionally effective [57,58], this is likely due to the significantly higher systemic exposure achieved by its active metabolite, ABZSO (Cmax: 1.30 μg/mL, AUC: 28.7 µg.h/mL; [59]). By comparison, the exposure levels for OFZ (Cmax: 0.64 μg/mL) and FBZ (Cmax: 0.14 μg/mL) observed here at 5 mg/kg fall below the threshold required to sufficiently inhibit tubulin polymerization [42], thereby limiting flukicidal activity.

To actively interact with its target receptor within the parasite, the drug must reach effective concentrations at the receptor site [60]. Previous studies involving various flukicidal compounds have shown that systemic concentrations are crucial for their activity, given that oral ingestion is the main route of drug accumulation into the fluke parasite [28–30]. Thus, the over five-fold increases in the plasma systemic availability of OFZ and its metabolites after administration of the 30 mg/kg experimental dose in sheep, could be relevant to achieve flukicidal efficacy. In ruminants, unlike monogastric animals, oral formulations reach the rumen, where they mix with and adsorb to particulate digesta shortly after administration [61]. This binding delays the drug´s passage through the gastrointestinal tract, promoting its particle dissolution in the abomasum’s acidic environment, ultimately enhancing drug systemic availability [24]. The OFZ administration at this high experimental oral dose (30 mg/kg) resulted in a greater amount of drug gradually passing into the abomasum, followed by absorption in the duodenum, which is consistent with the observed increase in OFZ absorption and systemic availability. Similar dose-dependent absorption has been reported for OFZ in goats [62] and ABZ in sheep [63,64]. Additionally, a positive correlation existed between doses and plasma and target tissue (liver, bile, and collected fluke parasites) concentrations for both active analytes, OFZ and FBZ (Table 2 and Fig 3). The liver exhibited the highest drug concentrations because it received the absorbed drug directly via the portal circulation. However, the drug may bind to liver tissue, making it unavailable to mature flukes residing in the bile ducts, canaliculi, or gallbladder [65]. The mature adult flukes in the bile ducts are exposed to high OFZ and FBZ concentrations present in both the plasma (via oral ingestion) and the bile (via transtegumental diffusion). The anthelmintic activity of FBZ is higher than that of OFZ, as supported by previous studies where FBZ showed greater potency in inhibiting the polymerization of bovine brain tubulin [66], and nematode motility in *ex vivo* studies [67]. Although the contribution of FBZ to the anthelmintic efficacy against *F. hepatica* after conventional treatment with OFZ (5 mg/kg) may be poorly relevant due to the low concentrations reached in the bloodstream, this contribution becomes more relevant after the administration of the high 30 mg/kg dose rate.

The current study did not specifically compare the efficacy of the different OFZ dose rates used against *F. hepatica.* We acknowledge as a limitation of the present study that non-equivalent blood and tissue sampling time points were used between the experimental groups (12 h for the 5 mg/kg dose and 24 h for the 30 mg/kg dose). This chronological difference in sampling was designed to significantly reduce the total number of experimental animals required for a full kinetics profile. Since each experimental group was strategically sampled at its respective expected peak concentration, which should coincide with its maximum drug exposure (accumulation) at the target host tissue, this methodological divergence must be explicitly considered when interpreting the dose-dependent accumulation for both the parent drug and its metabolites. Although these observations are not conclusive as an efficacy study, they suggest a strong correlation between systemic exposure and the impact/damage on the parasite. These results are also consistent with the data reported by Gomez-Puerta et al. [34], who observed a clear reduction in *F. hepatica* eggs in the feces of sheep treated with the same single oral dose of OFZ (30 mg/kg). Additionally, the work reported here is complementary to a clinical efficacy trial aimed at characterizing the flukicidal efficacy of OFZ at 30 mg/kg, which was performed in our Laboratory [57]. While these results confirm that OFZ displays dose-dependent flukicidal activity in sheep, its pharmacological potency remains significantly lower than that of albendazole (ABZ).

The data shown here demonstrate that the OFZ dose increment is associated with a higher plasma drug exposure and enhanced accumulation into the target parasite, which help to explain OFZ efficacy against adult liver flukes at 30 mg/kg dose. The pharmacological data reported here contribute to a clearer understanding of the close PK-PD relationship in flukicidal drug activity.

## Supporting information

S1 Table Raw pharmacokinetic data. Individual plasma and tissue concentrations of oxfendazole and its metabolites measured in sheep at each sampling time.
(XLSX)
